# Delay and restricted access of new molecules in Turkey compared to the United States and European Union

**DOI:** 10.3402/jmahp.v1i0.22799

**Published:** 2013-11-15

**Authors:** Toros Şahin, Atakan Yeşil, Türker Topcu

**Affiliations:** Sanofi Turkey, Market Access, Istanbul, Turkey

**Keywords:** launch, access to medicine, Turkish pharmaceutical market, new molecules

## Abstract

**Objective:**

This study compares the performances of new-molecule (NM) launches in Turkey with those in the European Union and United States for the years 2007–2013.

**Methods:**

The Thomson Reuters Newport Horizon for Innovators Database is used to identify NMs with a launch date after January 1, 2007, worldwide and marketing authorization approval after January 1, 2007, in the European Union. The launch dates for the European Union, the United States, and Turkey were retrieved from the same database. Data for Turkey were confirmed via IMS and RxMedia.

**Results:**

Out of 183 records identified that are launched in the European Union, the United States, or both, 44 of the NMs are launched in Turkey (24%).

**Conclusion:**

The results of this study show that 24% of the NMs that are launched in either the European Union or United States were able to be launched in Turkey with a mean delay of 821 days (2.25 years).

Turkey has undergone a broad health transformation program since 2003. The ultimate aim of the health transformation program was equity in access to healthcare for all citizens, as individuals with equal rights in the country (1). Starting with the implementation of the transformation program, regulations on the pharmaceutical market were also intensified, as a result of increasing access to available pharmaceuticals and the potential of increasing costs. The key and most important regulatory changes for the pharmaceutical industry during the period of 2003–2013 can be summarized as follows: (1) international reference pricing, which was started in 2004; (2) global budget cap application since 2009; and (3) good manufacturing practices (GMP) audit requirements for import drugs since 2009 (2). Healthcare expenditures per capita in Turkey have risen from 184 USD in 2002 to 640 USD in 2010, and they are expected to continue to grow considering the impacts of the continuing reforms. However, the reference pricing application since 2004 had a downwards price pressure on the pharmaceuticals. The rate of increase in drug consumption in volume was 24% on average during the 1995–2002 period, and it has increased to 125% during the 2002–2010 period. However, in comparison, the increase in the expenditure in value during 1995–2002 was 170%, whereas it was reduced to 21% during 2002–2010. This is generally attributed to the cost-containment and pricing policies applied by the regulators (3). Another important regulation aiming to control the prices of pharmaceuticals was the global budget cap application. According to this application, the regulators placed significant price cuts and discount increases on pharmaceuticals from the end of 2009 until the end of 2011. These price cuts, when combined with the negative impact of the difference between the fixed euro-to-TL conversion rate imposed by the Turkish Ministry of Health (MoH) (1.9595 TL/€) and the Central Bank of Turkey exchange rate (2.61 TL/€), led to pharmaceutical prices approximately 70% lower than the lowest EU prices (4). Another regulation imposed by the regulators was GMP audit requirements, which were compelled by MoH for the medicines produced and imported to Turkey from abroad. Accordingly, a GMP audit by the MoH to the site where the product will be produced has been mandatory since 2009 (5). The primary idea of this change was to protect the local generic producers from cheaper imported generics. However, as all products were subject to the application, new molecules (NMs) that were submitted for regulatory approval were hardly hit due to the low number of auditors at the MoH and the lack of prioritization for audits in the beginning. This change in the legislation has led to delays in the approval of marketing authorizations of new medicines. Accumulations of all these changes led to a more difficult NM launch atmosphere. Within this context, this study intends to compare the performances of NM launches in Turkey with those in the European Union and the United States for the years between 2007 and 2013.

## Methods

The Thomson Reuters Newport Horizon for Innovators Database is used to identify the NMs with a launch date after January 1, 2007, worldwide and marketing authorization approval after January 1, 2007, in the European Union. The database was accessed from August 20, 2013, to August 23, 2013. The launch dates for the European Union and United States were retrieved from the same database. Data for launch of the NMs in Turkey were also retrieved from the same database, and these dates were also confirmed by IMS Dataview Turkey monthly sales data and RxMedia Pharma (http://www.eczanet.com). Official reimbursement dates in Turkey were retrieved from official Social Security Institution announcements from 2007 to 2013.

## Results

The search yielded 210 NMs with worldwide launch dates and marketing authorization in the European Union after January 1, 2007. Sub-analyses lead to the exemption of 27 of the results, which were launched earlier than 2007 worldwide but appeared in the search results due to various reasons. Out of the remaining 183 records that were launched in the European Union, the United States, or both, 44 of the new molecular entities were launched in Turkey (24%). Out of 44 of the NMs launched in Turkey, 34 of them got official reimbursement approval (77%), whereas the rest seemed to be launched without reimbursement. The mean difference of launch dates between the latest launch country (either the European Union or United States) and Turkey is 821 days (2.25 years), and the median is 807 days (2.21 years).

## Discussion

The health transformation program applied in Turkey since 2003 has intensified the controls on prices, reimbursement conditions, and access of new products on the market. The results of this study show that 24% of the new medicines that are launched in either the European Union or United States were able to be launched in Turkey with a mean delay of 821 days (2.25 years). There might be several reasons for this level of access of NMs to the general market, such as company-based delays during registration processes, delays due to non-prioritization in GMP audit processes, no launch decisions due to mandatory low price levels, and delays due to reimbursement evaluation processes. However, it would be unfair to comment that Turkish patients cannot reach the remaining 76% of NMs that seem to be not yet launched in the retail market in Turkey. There is a certain pathway for patients to access these NMs (e.g. importing drugs via the Turkish Pharmacists Association, or TPA) that was designed for use in extraordinary situations; however, increasingly, this is becoming the primary pathway for access to new medicines for patients. As the knowledge and experience of patients and physicians regarding this kind of access pathway are limited, there is still room for progress for new product access to the market and whole population. Whether or to what extent patients are getting access to the other 76% of the NMs via the TPA pathway remains a topic for further research.
